# Health related quality of life and comorbidity. A descriptive analysis comparing EQ-5D dimensions of patients in the German disease management program for type 2 diabetes and patients in routine care

**DOI:** 10.1186/1472-6963-11-179

**Published:** 2011-08-02

**Authors:** Dominik Ose, Antje Miksch, Elisabeth Urban, Iris Natanzon, Joachim Szecsenyi, Cornelia Ursula Kunz, Tobias Freund

**Affiliations:** 1 Department of General Practice and Health Services Research, University Hospital of Heidelberg, Vosstrasse 2, D-69115 Heidelberg, Germany; 2 Institute of Medical Biometry and Informatics, University Hospital of Heidelberg, Im Neuenheimer Feld 305, D-69120 Heidelberg, Germany

## Abstract

**Background**

The co-occurance of multiple medical conditions has a negative impact on health related quality of life (HRQoL) for patients with type 2 diabetes. These patients demand for intensified care programs. Participation in a disease management program (DMP) for type 2 diabetes has shown to counterbalance this effect. However, it remains unclear which dimensions of HRQoL are influenced by the DMP. The aim of this study was to explore the HRQoL dimensions of patients with type 2 diabetes in the German DMP and patients in routine care (RC).

**Methods**

This analysis is part of a comparative evaluation of the German DMP for patients with type 2 diabetes. A questionnaire, including the HRQoL measure EQ-5D, was mailed to a random sample of 3,546 patients with type 2 diabetes (59.3% female). The EQ-5D dimensions were analyzed by grouping patients according to their participation in the German DMP for diabetes into DMP and RC.

**Results**

Compared to patients in DMP, patients in RC reported more problems for the dimensions mobility (P < 0.05), self care (P < 0.05) and performing usual activities (P < 0.01). Depending on the number of other conditions, remarkable differences for reporting "no problems" exist for patients with six or more comorbid conditions regarding the dimensions mobility (RC = 8.7%, DMP = 32.3%), self care (RC = 43.5%, DMP = 64.5%), usual activities (RC = 13.0%, DMP = 33.9%) and anxiety or depression (RC = 37.0%, DMP = 48.4%).

**Conclusion**

Patients participating in the German DMP for type 2 diabetes mellitus show significantly higher ratings of their HRQoL in the dimensions mobility, self care and performing usual activities compared to patients in RC. This difference can also be observed in patients with significant comorbidities. As these dimensions are known to be essential for diabetes care, the German DMP may contribute to improved care even for comorbid diabetes patients.

## Background

A special disease management program (DMP) for patients with type 2 diabetes has been in place in Germany since 2003. This primary care-based continuous program is accessible for all patients with type 2 diabetes insured within the statutory health insurance. Currently more than 3,200,000 patients with type 2 diabetes participate in the DMP. Important elements of this approach like evidence-based clinical guidelines or transfer between different levels of care are defined by a national expert group. In contrast to vendor-supported programs in the United States, general practitioners in small- to medium-sized practices have an important role in coordinating the care of enrolled patients. Previous evaluations of this program show positive results regarding quality of care and health-related quality of life [1-3].

Nevertheless, dealing with co-morbidity is an enormous challenge in the German DMP for type 2 diabetes. Up to 90 percent of enrolled patients suffer from one or more co-occuring medical conditions [4]. Comorbidity is demanding for both healthcare systems and patients. It implies complex clinical management and increasing health care costs [5-7] as well as impaired health-related quality of life (HRQoL). It is known that the presence of co-occuring medical conditions has a negative impact on HRQoL for patients with type 2 diabetes [8-13]. We could previously show that the German DMP for type 2 diabetes may help to counterbalance the negative effect of comorbidity on HRQoL [14]. However, it remains unclear which dimensions of HRQoL are influenced by DMP. Therefore the aim of this analysis was to assess differences in the five dimensions of a valid multi-dimensional instrument for HRQoL (EQ-5D) between patients participating in the German DMP for type 2 diabetes and patients in routine care (RC).

## Methods

This analysis was performed as part of the ELSID study (Evaluation of a Large-scale Implementation of Disease management programs; 2005-2007). This observational study aims to compare the care provided within the DMP with routine care (RC). The study protocol was approved by the ethics committee of the University of Heidelberg [15].

All of the participants in this study were insured by 1 large statutory regional healthcare fund called the Allgemeine Ortskrankenkasse (AOK), which covers about 40% of the German population. Patients were identified from routine claims data of this healthcare fund. To be included in the study, patients had to be older than age 50 years and be receiving a prescription for antidiabetic medication (oral antidiabetic drugs or insulin) in the first half-year of 2005. Patients in the DMP group had to be enrolled in the program by December 31, 2005, regardless of how long they had participated in the program prior to that date. Patients in the non-DMP group were not enrolled in the DMP before this appointed date.

Overall n = 20,625 patients (59.2% female) were included in the ELSID-study. The population for the presented survey was a random sample of 3,546 patients (59.3% female) from all study patients. In 2006, these patients received questionnaires with a cover letter sent by their health insurance provider. Details of the data acquisition have already been published [16].

In this survey we used the EQ-5D, a validated generic instrument for measuring HRQoL, which is available in more than 50 languages. The self-report questionnaire consists of a descriptive system, which defines health in terms of five dimensions: mobility, self-care, usual activities, pain or discomfort, and anxiety or depression. Each dimension is divided into three levels, indicating no problem (1st level), some or moderate problems (2nd level) and extreme problems (3rd level). The level of problem, reported on each of the EQ-5D dimensions, determines a unique health state [17,18]. Further investigations have demonstrated the usefulness of EQ-5D in identifying determinants of health states [19,20]. The questionnaire also included questions on sociodemographic characteristics (age, gender, educational level, marital status, and household income), self-reported health information (weight, height, and smoking status) and a list of chronic conditions in lay language (hypertension, osteoarthrosis, cancer, previous stroke, coronary heart disease, COPD, asthma, heart failure, and previous heart attack).

The EQ-5D dimensions were analyzed by grouping patients according to their participation in the German DMP for diabetes into DMP and RC. To analyze differences between groups we compared the proportion of reported problems (none, moderate and extreme) and calculated chi-square tests for each dimension. To describe differences depending on comorbidity we compared the proportion of reporting "no problems" for each dimension between patient groups with different numbers of other conditions (0, 1, 2, 3, 4, 5, 6 and more). All analyses were conducted using SPSS (version 15.0).

## Results

A total of 1,532 questionnaires were returned (response rate: 42.2%). Valid data were available for 1,399 patients, more precisely 865 patients in DMP (61,83%) and 534 (38,17%) patients in RC. 53.6% of these were female and the mean age for the entire sample was 70.3 (±8.5) years. Significant differences between the two groups (DMP and RC) did not exist for the total sample but for some subgroups (Table 1).

**Table 1:** Patient characteristics

			Number of other chronic conditions													
	**Sample**		**no**		**1**		**2**		**3**		**4**		**5**		**6 **≤	

	DMP	RC	DMP	RC	DMP	RC	DMP	RC	DMP	RC	DMP	RC	DMP	RC	DMP	RC

N	865	534	66	40	189	111	214	140	178	91	93	64	60	42	65	46

Age (mean)	70.2	70.5	72.4	68.6*	69.2	69.6	69.8	71.3	70,4	70,9	69.5	70.8	71.8	70.7	72.2	71.8

Age (SD)	±8.3	±8.9	±8.0	±7.7	±8.0	±9.3	±8.5	±8.5	±8,2	±8,9	±7.6	±7.8	±9.0	±10.4	±8.9	±8.7

Female subjects (%)	53.8	53.4	53.0	55.0	55.6	47.7	57.9	58.9	51,7	59,3	47.3	50.0	56.7	40.5	44.6	56.5

Education ≤9 years (%)	70.8	72.3	76.7	81.1	75.9	75.5	75.0	79.6	74,7	85.7*	76.1	76.7	76.8	86.1	79.7	79.1

Living in partnership (%)	65.7	62.9	55.4	52.6	64.9	64.2	61.9	62.0	66,7	52.8*	63.7	63.5	52.6	61.9	64.1	51.1

BMI (mean)	30.3	30.3	29.1	30.3	29.4	30.2	30.5	29.3	30,7	30,4	31.3	30.1	31.0	33.2	30.2	31.4

BMI (SD)	±5.8	±6.5	±5.2	±5.2	±5.0	±6.1	±5.7	±6.1	±6,6	±7,5	±5.7	±5.7	±6.6	±7.4	±6.0	±7.2

Hypertension (%)	71.3	72.1	------	------	24.3	20.7	62.1	63.6	74,2	73,6	77.4	82.8	91.7	69.0	87.7	91.3

Osteoarthrosis (%)	57.2	56.7	------	------	57.7	49.5	79.0	77.9	83,7	89,0	87.1	90.6	85.0	90.5*	89.2	95.7

Coronary heart (%)	20.9	20.4	------	------	0.5	7.2*	7.0	7.1	24,2	17,6	50.5	42.2	51.7	40.5	67.7	67.4

Heart failure (%)	16.9	18.0	------	------	3.7	2.7	9.3	5.7	17,4	13,2	32.3	32.8	48.3	50.0	44.6	67.4*

COPD (%)	9.9	10.9	------	------	0.5	3.6*	4.2	3.6	10,7	9,9	25.8	25.0	13.3	16.7	38.5	37.0

Cancer (%)	7.6	9.0	------	------	3.2	1.8	5.6	4.3	6,7	11,0	16.1	17.2	13.3	4.8	20.0	37.0

Previous heart attack (%)	7.1	8.2	------	------	0.5	0.0	0.9	6.4*	8,4	8,8	18.3	10.9	11.7	19.0	29.2	26.1

Previous stroke (%)	6.0	6.6	------	------	1.1	2.7	3.7	2.1	9,0	7,7	6.5	12.5	15.0	11.9	16.9	19.6

Asthma (%)	4.2	4.5	------	------	0.0	0.9	1.4	1.4	7,3	2,2	4.3	4.7	11.7	26.2	13.8	10.9

* P < 0.05

The EQ-5D was completed by 1,291 patients. The analysis of EQ-5D dimensions showed significant differences for reporting problems in the dimensions mobility (P < 0.05), self care (P < 0.05) and performing usual activities (P < 0.01). For the dimensions pain or discomfort and anxiety or depression we found no significant difference (Table 2). Depending on the number of co-occuring conditions differences for reporting "no problems" could be observed particularly for patients with six or more co-occuring conditions in the dimensions mobility (RC = 8.7%, DMP = 32.3%), self care (RC = 43.5%, DMP = 64.5%), usual activities (RC = 13.0%, DMP = 33.9%) and anxiety or depression (RC = 37.0%, DMP = 48.4%) (Table 3). However, these observed differences were not statistically significant.

**Table 2:** Analysis of EQ-5D dimensions (n = 1291)

Dimension	Sample		Problems			p-value*
	RC	DMP	level	RC	DMP	

	n	n		%	%	

mobility	482	809	no	51,3	54,6	0.041

			some	47,6	45,2	

			extreme	1,0	0,1	

self care	482	809	no	80,1	84,7	0.010

			some	15,7	13,7	

			extreme	4,1	1,6	

performing usual activities	482	809	no	56,3	59,5	0.006

			some	35,0	36,2	

			extreme	8,7	4,3	

pain/discomfort	482	809	no	19,0	19,4	0.088

			some	65,6	69,5	

			extreme	15,3	11,1	

anxiety/depressed	482	809	no	67,5	66,9	0.682

			some	28,4	29,8	

			extreme	4,1	3,3	

*chi-square test						

**Table 3:** No problems reported by number of other chronic conditions (n = 1291)

number of other conditions	sample		mobility		self care		usual activities		pain/discomfort		anxiety/depression	
	
	RC	DMP	RC	DMP	RC	DMP	RC	DMP	RC	DMP	RC	DMP
	n	n	%	%	%	%	%	%	%	%	%	%

**0**	32	58	87,5	75,9	90,6	86,2	71,9	86,2	40,6	55,2	87,5	82,8

**1**	99	179	75,8	74,9	91,9	93,9	77,8	82,1	37,4	36,9	80,8	76,0

**2**	129	203	56,2	51,7	83,1	85,2	60,8	59,1	20,8	14,8	70,8	66,5

**3**	82	163	50,0	53,4	79,3	85,9	58,5	52,8	11,0	9,8	63,4	62,0

**4**	58	86	29,3	38,4	81,0	81,4	46,6	47,7	5,2	9,3	65,5	65,1

**5**	36	58	27,8	32,8	75,0	75,9	33,3	27,6	5,6	3,4	52,8	60,3

≥**6**	46	62	8,7	32,3	43,5	64,5	13,0	33,9	2,2	4,8	37,0	48,4

## Discussion

The analysis revealed differences between DMP and RC patients for the HRQoL dimensions "mobility", "self care", and "performing usual activities". DMP patients reported significantly less problems in these dimensions. These differences could also be observed in patients with significant comorbidities.

These results are in line with our previous findings [14] and provide additional understanding of how the German DMP for patients with type 2 diabetes mellitus may improve HRQoL. However, methodological limitations of descriptive studies do not allow any conclusion on causal relationship. Nevertheless, the results give rise to the question as to which elements of the German DMP for type 2 diabetes could be contributing to the differences between groups. Indeed, we know that aspects like physical activities or social support can improve HRQoL [21-23]. Even so, it is uncertain which specific elements of DMP, such as structured care, regular follow ups or patient education programs, are responsible for the fact that patients - especially those with numerous other conditions - reported less problems.

Physical activity is an essential component of diabetes management [24]. Therefore, reporting less problems in the HRQoL dimension "mobility" may indicate that a higher proportion of DMP patients would be able to be physically active than RC patients. However, the actual level of physical activity of both groups was not assessed in this survey. "Self-care" as well as the ability to perform "usual activities" is seen to be crucial for independent living and HRQoL. The observed differences may therefore explain our previous results [14].

Our study is further limited by a moderate response rate. This rate might have been higher if the questionnaires had been sent out by the university department directly instead of the health fund. Due to a strict protection of data privacy we were not able to contact the patients directly. Also we do not know whether and how motivation to participate in a DMP affects HRQoL. Potential differences (age, gender, DMP status) between responders and non-responders may also affect our results. It should be considered that the age of patients in our sample is substantially higher than usually seen in diabetes studies.

## Conclusions

Patients participating in the German DMP for type 2 diabetes mellitus show significantly higher ratings of their HRQoL in the dimensions mobility, self care and performing usual activities compared to patients in RC. This difference can also be observed in patients with significant comorbidities. As these dimensions are known to be essential for diabetes care, the German DMP may contribute to improved care even for comorbid diabetes patients.

## Competing interests

The authors declare that they have no competing interests.

## Authors' contributions

AM and JS initiated, designed and coordinated the study. DO and TF carried out data-analysis and wrote the manuscript. CUK gave statistical advice. All authors, particularly IN and EU, read earlier versions of the manuscript, provided critical comments, and approved the final manuscript.

## Pre-publication history

The pre-publication history for this paper can be accessed here:

http://www.biomedcentral.com/1472-6963/11/179/prepub
